# Physiological Arousal During Alcohol Cue Exposure in Adult Drinkers: Sex Differences in Neural and Clinical Correlates: Alcohol Cue Exposure and Arousal

**DOI:** 10.1111/adb.70197

**Published:** 2026-11-03

**Authors:** Yu Chen, Hak Kei Wong, Shefali Chaudhary, Herta H. Chao, Sheng Zhang, Chiang‐Shan R. Li

**Affiliations:** ^1^ Department of Psychiatry Yale University School of Medicine New Haven Connecticut USA; ^2^ University College London London UK; ^3^ Department of Medicine VA Connecticut Healthcare System West Haven Connecticut USA; ^4^ Department of Medicine Yale University School of Medicine New Haven Connecticut USA; ^5^ Department of Neuroscience Yale University School of Medicine New Haven Connecticut USA; ^6^ Inter‐Department Neuroscience Program Yale University New Haven Connecticut USA; ^7^ Wu Tsai Institute Yale University New Haven Connecticut USA

**Keywords:** alcohol cue, anterior cingulate, drug cue, galvanic skin response, MRI

## Abstract

Cue exposure contributes to craving and reinforces alcohol misuse, potentially by altering interoceptive processing and physiological arousal. However, few studies have reported changes in physiological arousal or their neural underpinnings in response to alcohol cues. Here, we addressed these questions in 94 adult drinkers (39 females; age mean ± SD: 59.1 ± 19.1 years and range: 19–85 years) who underwent functional magnetic resonance imaging and skin conductance recording during alcohol and neutral cue exposures and completed clinical assessments including the Alcohol Use Disorder Identification Test (AUDIT; mean ± SD: 4.4 ± 5.5). Imaging data were processed with published routines and evaluated at a corrected threshold. The results showed significantly higher skin conductance response in ‘alcohol>neutral’ blocks (SCR_Al‐Ne_) in females than in males. However, SCR_Al‐Ne_ was positively correlated with participants' AUDIT scores in males but not in females. SCR_Al‐Ne_ was also positively correlated with global positive alcohol expectancy and novelty seeking scores in males but not in females. A voxel‐wise two sample test of the contrast ‘alcohol>neutral’ did not show any clusters with significant sex differences in cue reactivity. Whole‐brain regression of the same contrast on AUDIT scores identified a large cluster in the perigenual anterior cingulate cortex (pgACC), with the beta estimates showing a significant positive correlation with the SCR_Al‐Ne_, again in males only. These sex differences were all confirmed by slope tests. Thus, males with more drinking problems showed higher arousal as well as the pgACC response to alcohol cues in association with alcohol expectancy and novelty seeking. In chain mediation modelling, the pgACC response and alcohol expectancy interlinked SCR_Al‐Ne_ and AUDIT scores in males. In contrast, we failed to observe any significant neural or psychological correlates of physiological arousal, despite higher SCR_Al‐Ne_, in females. With the caveat of a smaller sample size for females, these findings highlight sex and individual differences in physiological responses to alcohol cues and the neural and psychological underpinnings in male adult drinkers. Whether or how physiological arousal may play a role in the pathophysiology of alcohol misuse remains to be investigated for females.

## Graphical abstract

In 94 adult drinkers, females exhibited greater physiological arousal to ‘alcohol〉neutral’ cues, but only males showed positive associations between cue‐evoked arousal and alcohol expectancy, novelty seeking, pgACC response and problem drinking severity. In males, pgACC response and alcohol expectancy mediated the relationship between physiological arousal and problem drinking severity. These findings highlight potential sex differences in the physiological and neural correlates of alcohol cue reactivity, a finding warranting replication in larger, more balanced samples.

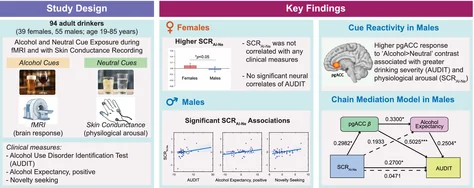

## 1 Introduction

### 1.1 Alcohol Cue Exposure Contributes to Drinking

Cue exposure reinforces alcohol misuse [1]. Through repeated pairing alcohol consumption with visual, social, environmental and other contextual stimuli, these cues acquire motivational salience and become associated with alcohol‐related reward and reinforcement [1]. As a result, exposure to alcohol cues can elicit conditioned responses, including craving and approach behaviours, even in the absence of alcohol availability [2]. By triggering these responses, alcohol‐related cues may increase the likelihood of alcohol seeking and consumption and thereby contribute to the maintenance and escalation of drinking. In laboratory studies, exposure to alcohol cues evokes craving [3] and increases alcohol consumption [4], supporting a central role of cue reactivity in the development and persistence of alcohol misuse.

Imaging studies have consistently shown that alcohol cue exposure engages a distributed network of brain regions, encompassing the medial prefrontal cortex (mPFC), anterior cingulate cortex (ACC), striatum, amygdala, hippocampus and insula. Meta‐analytic evidence further indicates that these regions constitute a core neural circuitry underlying alcohol cue reactivity and craving [5, 6]. Collectively, these areas are implicated in the psychological processes critical to alcohol and substance misuse, including reward valuation, salience attribution, memory and action selection [7]. Importantly, individual differences in cue‐elicited neural responses have been linked to alcohol expectancy, drinking severity and relapse risks [8, 9, 10]. However, this literature has focused primarily on subjective craving and neural responses to alcohol cues, with comparatively less attention devoted to physiological arousal as a component of cue reactivity.

### 1.2 Changes in Physiological Arousal During Alcohol Cue Exposure

Physiological arousal reflects interoceptive processing and represents a core component of cue reactivity [11]. Addiction models propose that repeated alcohol use sensitizes neural systems involved in assigning motivational salience to alcohol‐related stimuli, resulting in heightened attentional capture, craving and physiological arousal during cue exposure [12]. In these models, alcohol cues trigger both autonomic and subjective responses through interactions among salience, reward and interoceptive processing systems. Skin conductance response (SCR), which results from sympathetic nervous system activation, provides a physiological measure of motivational salience of alcohol cues [13, 14]. Physiological arousal, as manifested in SCR, may influence craving and decision‐making [15] in alcohol misuse. Brain regions such as the insula, ACC and mPFC are thought to integrate autonomic signals with motivational and cognitive processes, thereby translating bodily arousal into subjective urge and goal‐directed behaviours [16, 17]. The insula is particularly important for integrating bodily states with conscious awareness of craving, whereas the ACC contributes to salience detection and behavioural adaptation [18]. Through these mechanisms, SCR may capture an early and relatively automatic stage of cue reactivity that reflects the processing of motivationally salient alcohol cues before conscious craving emerges. Thus, alcohol cue‐elicited SCR may index automatic physiological responses that precedes conscious craving and contributes to drinking without one's full awareness.

SCR has been widely used to index stimulus‐evoked arousal across a wide variety of behavioural contexts, including cognitive control, fear conditioning, negative emotion processing, reward learning and substance use [19]. Because SCR reflects autonomic responses to salient stimuli, it has become an important physiological marker in cue‐reactivity research. However, the findings on SCR to alcohol cues are mixed, with some studies showing elevated cue‐elicited SCR [20, 21] whereas others reporting no differences in social drinkers [22] or in patients with alcohol dependence [23]. Notably, in a study of individuals with alcohol use disorder (AUD), those with lower drinking severity exhibited greater anticipatory SCRs prior to making decisions in the Iowa Gambling Task (IGT), whereas individuals with more severe AUD did not show this physiological response [24]. Although the latter study did not involve alcohol‐related cues, decision‐making in the IGT could be highly arousing, and the lack of increases in SCR in people with more severe AUD suggests deficits in interoceptive processing, which may impact the manifestation of physiological arousal during cue exposure. Together, these findings suggest that cue‐elicited SCR can differ among people with varying levels of drinking severity. This variability, along with the noisy nature of SCR and its dependence on individuals' behavioural traits [25], may account for the mixed literature. No studies to our knowledge have systematically evaluated the impact of individual behavioural traits on physiological arousal in response to alcohol cues.

### 1.3 Sex Differences in Alcohol Cue Reactivity

Sex differences have been reported in subjective and neural responses to cue exposure [26]. For instance, craving appeared more closely associated with heightened arousal following stress and alcohol cues in males than in females among social drinkers [27]. Male relative to female patients with alcohol dependence showed stronger striatal cue responses during functional magnetic resonance imaging (fMRI; [28]). A more recent study found that in females but not males with AUD, higher craving was associated with the hypoactivation of bilateral dorsolateral prefrontal cortex in response to alcohol as compared to neutral cues. In contrast, greater hippocampal activation predicted more heavy drinking days during treatment in males only [29]. Together, these findings suggest that females and males may engage distinct neural circuits during the processing of alcohol‐related cues, with potential sex differences in the integration of salience‐related and interoceptive signals that contribute to craving and cue reactivity [30]. On the other hand, no studies have reported sex differences in physiological arousal in response to alcohol cues or addressed sex differences in the impact of behavioural traits on cue‐elicited arousal.

### 1.4 The Present Study

In the present study, we characterized physiological arousal during alcohol cue exposure and examined its psychological/behavioural and neural correlates, with a particular focus on sex differences. Adult drinkers were exposed to alcohol and neutral cues in alternating blocks during fMRI and concurrent recording of skin conductance. We quantified cue‐elicited physiological arousal as the difference in SCR between alcohol and neutral blocks (SCR_Al‐Ne_) and evaluated their associations with the severity of problem alcohol use, alcohol expectancy, novelty seeking and neural responses to ‘alcohol>neutral’ cues. Based on prior work implicating medial prefrontal and cingulate regions in cue salience and interoceptive processing, we hypothesized that cue‐elicited physiological arousal would be associated with these regional responses and that the strength and psychological correlates of these associations would differ by sex. Our goal was to elucidate sex differences in alcohol cue reactivity specifically with respect to physiological arousal and the neural and psychological correlates of the sex differences.

## 2 Methods

### 2.1 Participants and Assessments

Ninety‐four adults (39 females; 19–85 years) recruited from the Greater New Haven area of Connecticut, United States, participated in this study. As described in our earlier work [31], the inclusion/exclusion criteria included (1) consumption of at least one alcoholic drink in the past year; (2) physically healthy, with no major medical conditions and scores ≥ 27 in Mini Mental State Examination; (3) those with current use of prescription medications or with a history of head injury or neurological illness were excluded; and (4) those who met criteria of current or history of Axis I disorders (except AUD) according to the Structured Clinical Interview for DSM‐IV [32] or reported current use of illicit substances or tested positive for cocaine, methamphetamine, opioids, marijuana, barbiturates or benzodiazepines were excluded. Note that 16 participants (5 females) met DSM‐IV criteria for AUD in the current sample. In addition, 9 participants reported current cigarette smoking and had the scores of Fagerström Test for Nicotine Dependence less than 6. The Human Investigation Committee at Yale School of Medicine approved the study procedures. All participants signed an informed consent prior to the study.

All participants completed assessments with the Alcohol Use Disorder Identification Test (AUDIT; [33, 34]), Alcohol Expectancy Questionnaire (AEQ; [35]) and Tridimensional Personality Questionnaire (TPQ; [36]) and reported years of drinking, binge drinking months per year and drinking days per month during last year.

AUDIT assesses the frequency of alcohol consumption, dependence and associated problems, with a higher score indicating greater severity of problem alcohol use. AEQ evaluates individual expectancies of the effects of drinking. With all subscores of the AEQ highly correlated [35], we focused here on the global positive subscore or AEQ‐GP, which reflects expectation of physical (e.g., relaxation) and psychological (e.g., sociability) effects from drinking, in data analyses. The TPQ addresses reward expectancy, harm avoidance and novelty seeking (NS). We focused on the NS subscore or TPQ‐NS, which is implicated in alcohol misuse [37], in our analyses.

### 2.2 Cue‐Induced Alcohol Craving Task

As described in an earlier study [38], participants were engaged in a cue‐induced alcohol craving task, with 12 alternating blocks of alcohol and neutral images, during fMRI. Specifically, a cross was used to engage attention at the beginning of each block. After 2 s, six pictures displaying alcohol‐related cues (alcohol blocks) or neutral cues (neutral blocks) were shown for 6 s each. Participants were asked to view and ponder how they might relate to the images. The images were collected from the Internet and independently reviewed by two investigators. Alcohol pictures included bar scenes, individuals or a group of people holding or drinking alcoholic beverages and images of a variety of alcoholic drinks, such as beer, wine and vodka. Neutral pictures depicted nonalcoholic drinks or people in a social setting without alcohol consumption. Participants were asked at the end of each block to report how much they craved for alcohol with rating from 0 (no craving at all) to 10 (highest craving ever experienced) on a visual analogue scale. Each block lasted about 45 s, including time for craving rating. A total of six alcohol and six neutral blocks took approximately 9 min to complete. Each participant completed one run of the task. The differences in craving rating between alcohol and neutral blocks were computed as CR_Al‐Ne_ for further analyses.

### 2.3 Imaging Protocol, Data Preprocessing and Modelling

Conventional T1‐weighted spin echo sagittal anatomical images were acquired for slice localization using a 3T scanner (Siemens Trio). Anatomical images of the functional slice locations were next obtained with spin echo imaging in the axial plane parallel to the AC‐PC line with repetition time (TR) = 1900 ms, echo time (TE) = 2.52 ms, bandwidth = 170 Hz/pixel, field of view (FOV) = 250 × 250 mm, matrix = 256 × 256, 176 slices with slice thickness = 1 mm and no gap. Functional blood oxygenation level‐dependent (BOLD) signals were acquired using multiband imaging (multiband acceleration factor = 3) with a single‐shot gradient echo echoplanar imaging sequence. Fifty‐one axial slices parallel to the anterior‐posterior commissure line covering the whole brain were acquired with TR = 1000 ms, TE = 30 ms, bandwidth = 2290 Hz/pixel, flip angle = 62°, FOV = 210 × 210 mm, matrix = 84 × 84, with slice thickness = 2.5 mm and no gap. Imaging data were preprocessed using SPM12 (Wellcome Centre for Human Neuroimaging). No BOLD runs were removed due to significant motion (> 3‐mm translation peak‐to‐peak movement and/or 1.5° rotation). Images from the first five TRs at the beginning of each run were discarded to ensure only BOLD signals at steady‐state equilibrium between radio frequency pulsing and relaxation were included in analyses. Images of each subject were first realigned (motion corrected) and corrected for slice timing. A mean functional image volume was constructed for each subject per run from the realigned image volumes. These mean images were co‐registered with the high‐resolution structural image and then segmented for normalization with affine registration followed by nonlinear transformation. The normalization parameters determined for the structure volume were then applied to the corresponding functional image volumes for each subject. Images were resampled to 3‐mm isotropic voxel size. Finally, the images were smoothed with a Gaussian kernel of 4‐mm full width at half maximum.

We employed general linear model (GLM) to identify regional responses to ‘alcohol>neutral’ cues. For each subject, alcohol and neutral cue blocks were modelled as separate regressors using boxcar functions corresponding to block onset and duration and convolved with the canonical hemodynamic response function. A high‐pass temporal filter (i.e., 128‐s cutoff) was applied to remove low‐frequency drifts, and serial correlations were accounted for using a first‐order autoregressive model. We distinguished alcohol and neutral blocks for each subject and included the realignment parameters in all six dimensions in the GLM. The GLM was used to estimate the component of variance explained by each regressor. We constructed for each subject the contrast of ‘alcohol>neutral’ blocks to evaluate regional activities that differentiated viewing of alcohol and neutral cues.

### 2.4 Skin Conductance Response: Data Acquisition and Analysis

With a Biopac MP150 system, skin conductance was continuously recorded during fMRI from the palmer surfaces of the index and middle fingers of the left hand. The Biopac system used AcqKnowledge 4.1 software (Biopac Systems, USA) and the Biopac electrodermal activity amplifier module (Galvanic Skin Response [GSR] 100c) set at a channel sampling rate of 31 Hz and a gain of 5 μSiemens (μS) per volt (resulting in a resolution of 0.0015 μS). The recording of skin conductance was synchronized with the behavioural task and image acquisition. A smoothing function with a moving average of 500 ms was applied in order to eliminate high‐frequency noise [39]. We used the entire block (36‐s window) to compute the SCR associated with each block. Thus, the SCR of each block was defined as the onset‐to‐peak amplitude difference in skin conductance in this 36‐s window [40]. Specifically, we computed the SCR to alcohol and neutral cues by subtracting the level of skin conductance over the entire 36 s (six picture cues each presented for 6 s) from the baseline, defined as the signal at time = 0 s. SCR recordings were visually inspected for signal quality before analysis. No data exhibited excessive noise, signal dropout, or recording failure that required exclusion. Because the primary objective was to quantify cue‐evoked responses within each block, habituation across repeated cue blocks was not formally evaluated.

### 2.5 Statistical Analyses

#### 2.5.1 Clinical and Physiological Data Analyses

We first performed two‐sample *t*‐tests to examine sex differences in age, AUDIT, AEQ‐GP and TPQ‐NS scores, as well as CR_Al‐Ne_. We computed and compared SCR during ‘alcohol> neural’ cue exposure (SCR_Al‐Ne_) in males and females separately and performed paired‐sample *t*‐tests to examine condition differences in each sex. We tested sex differences in the within‐subject contrast of SCR_Al‐Ne_ in an analysis of variance (ANOVA) with age as a covariate. For the intersubject variation in SCR_Al‐Ne_, we performed linear regression against AUDIT, AEQ‐GP and TPQ‐NS scores, as well as CR_Al‐Ne_ in males and females separately with age as a covariate and examined sex differences in the correlation using slope tests [41]. The behavioural analyses were conducted to evaluate a priori hypotheses regarding associations between cue‐elicited responses and alcohol‐related behavioural measures. Because these analyses were prespecified rather than exploratory, formal correction for multiple comparisons was not applied. Nonetheless, findings with modest effect sizes or marginal *p* values should be interpreted cautiously.

#### 2.5.2 Imaging Data Analyses: One Sample *t*‐Tests and Whole‐Brain Regression

For the imaging data, we first employed a one‐sample *t*‐test to evaluate voxel‐wise brain response to cue exposure in a contrast of ‘alcohol>neutral’ for all, males and females alone and evaluated the results at voxel level *p* < 0.001, uncorrected in combination with cluster level *p* < 0.05, family‐wise error rate (FWE) corrected, with a minimum cluster size of *k* = 18. The same threshold was used to evaluate the results of all following analyses. We examined sex differences in brain responses to ‘alcohol>neutral’ cues by a two‐sample *t*‐test, with age as a covariate. Next, we performed whole‐brain regression on brain responses to the same contrast against AUDIT, AEQ‐GP and TPQ‐NS scores, as well as CR_Al‐Ne_, with age as a covariate, across all subjects and in males and females separately. We also performed supplementary whole‐brain regression analyses examining alcohol and neutral cue responses in link with clinical measures.

For clusters identified from whole‐brain regressions that were in significant correlation with any clinical measures in males or in females alone, we performed region‐of‐interest (ROI) analyses by estimating *β* values of regional activation, computing the partial correlations of *β*'s with clinical measures in each sex and following up with slope tests to examine sex differences in the correlations, all with age as a covariate. As noted in our prior work [42], these slope tests did not represent ‘double‐dipping,’ as the regression maps were identified with a threshold and clusters showing significant correlations in males might have just missed the threshold in females and vice versa. Thus, direct tests of the slopes of regression were needed to confirm sex differences.

#### 2.5.3 Chain Mediation Analyses

Given the AUDIT, AEQ‐GP scores, regional activity *β* in the perigenual ACC (pgACC) and SCR_Al‐Ne_ were significantly intercorrelated in males alone (see Results), we tested a series of chain mediation models in which each variable was alternately specified as the predictor, mediator(s) or outcome. Specifically, we examined whether the associations between the predictor (X) and outcome (Y) were sequentially mediated by two intervening variables (M1 and M2). Indirect effects were estimated using a nonparametric bootstrapping procedure with 5,000 resamples and 95% bias‐corrected confidence intervals. Age was included as a covariate in all regression equations. The significance of indirect effects was determined by whether the bootstrap confidence intervals excluded zero. Both total and direct effects were also estimated to characterize the overall and residual associations between X and Y.

## 3 Results

### 3.1 Clinical Measures of the Sample

Demographic and clinical metrics are shown separately for males and females in Table 1. With age as a covariate, males and females did not differ in AUDIT (*F* = 1.08, *p* = 0.303), AEQ‐GP (*F* = 0.26, *p* = 0.612) or TPQ‐NS (*F* = 0.26, *p* = 0.611) scores. The paired sample *t*‐test showed that craving rating was significantly higher in alcohol (1.91 ± 1.58) relative to neutral (1.28 ± 0.80) blocks (*t* = 6.02, *p* = 3.5 × 10^−8^) across all subjects. Furthermore, males and females showed a small but significant difference in CR_Al‐Ne_ (males = 0.78 ± 1.16 vs. females = 0.43 ± 0.78; *F* = 4.50, *p* = 0.037, one‐way ANOVA with age as a covariate).

**TABLE 1:** Demographic and clinical measures of males and females.

	Males (*n* = 55)	Females (*n* = 39)	*t*/*F*	*p*
Age (years)	61.0 ± 16.4	56.6 ± 22.4	1.05	0.300
Years of drinking	34.5 ± 19.3	44.4 ± 19.0	−2.40	0.018
Binge drinking months/year	1.7 ± 5.5	0.4 ± 1.0	1.66	0.102
Drinking days/month last year	7.5 ± 9.7	10.0 ± 9.8	−1.25	0.215
AUDIT	4.8 ± 5.9	3.8 ± 4.8	1.08	0.303
AEQ‐GP	11.6 ± 5.2	11.5 ± 5.3	0.26	0.612
TPQ‐NS	3.3 ± 2.5	3.6 ± 2.5	0.26	0.611
CR_Al‐Ne_	0.78 ± 1.16	0.43 ± 0.78	4.50	0.037

*Note:* Values are mean ± SD A two‐sample *t*‐test was performed for age and one‐way ANOVA tests for clinical measures (with age as a covariate).

Abbreviations: AEQ, Alcohol Expectancy Questionnaire; AUDIT, Alcohol Use Disorders Identification Test; CR_Al‐Ne_, craving ratings during ‘alcohol>neutral’ blocks; GP, positive global changes in experience; NS, novelty seeking; TPQ, Tridimensional Personality Questionnaire.

With age as a covariate, CR_Al‐Ne_ was significantly correlated with AUDIT scores both in males (*r* = 0.42, *p* = 0.002) and in females (*r* = 0.45, *p* = 0.005), with no significant sex difference (slope test: *z* = 0.17, *p* = 0.865). Across all subjects, CR_Al‐Ne_ was significantly correlated with AUDIT scores (*r* = 0.43, *p* = 1.4 × 10^−5^). Males showed significant correlations of AEQ‐GP scores with both AUDIT scores (*r* = 0.46, *p* = 4.5 × 10^−5^) and CR_Al‐Ne_ (*r* = 0.36, *p* = 0.008). Females showed a significant correlation of AEQ‐GP scores with AUDIT scores (*r* = 0.36, *p* = 0.027) but not with CR_Al‐Ne_ (*r* = 0.29, *p* = 0.083). Males showed a significant correlation of TPQ‐NS scores with AUDIT scores (*r* = 0.30, *p* = 0.029) but not with CR_Al‐Ne_ (*r* = 0.07, *p* = 0.632), whereas females did not show significant correlations of TPQ‐NS scores with AUDIT scores (*r* = 0.15, *p* = 0.365) or with CR_Al‐Ne_ (*r* = 0.07, *p* = 0.657). Scatter plots of the data points in residuals along with the regression lines and 95% confidence intervals are shown in Figure 1. However, slope tests with age as a covariate did not confirm significant sex differences in any of these correlations (*p's* ≥ 0.465). Across all subjects, AEQ‐GP scores were significantly correlated with AUDIT scores (*r* = 0.43, *p* = 1.5 × 10^−5^) and CR_Al‐Ne_ (*r* = 0.34, *p* = 0.001), while TPQ‐NS scores were significantly correlated with AUDIT scores (*r* = 0.23, *p* = 0.024) but not with CR_Al‐Ne_ (*r* = 0.05, *p* = 0.636).

**FIGURE 1:**
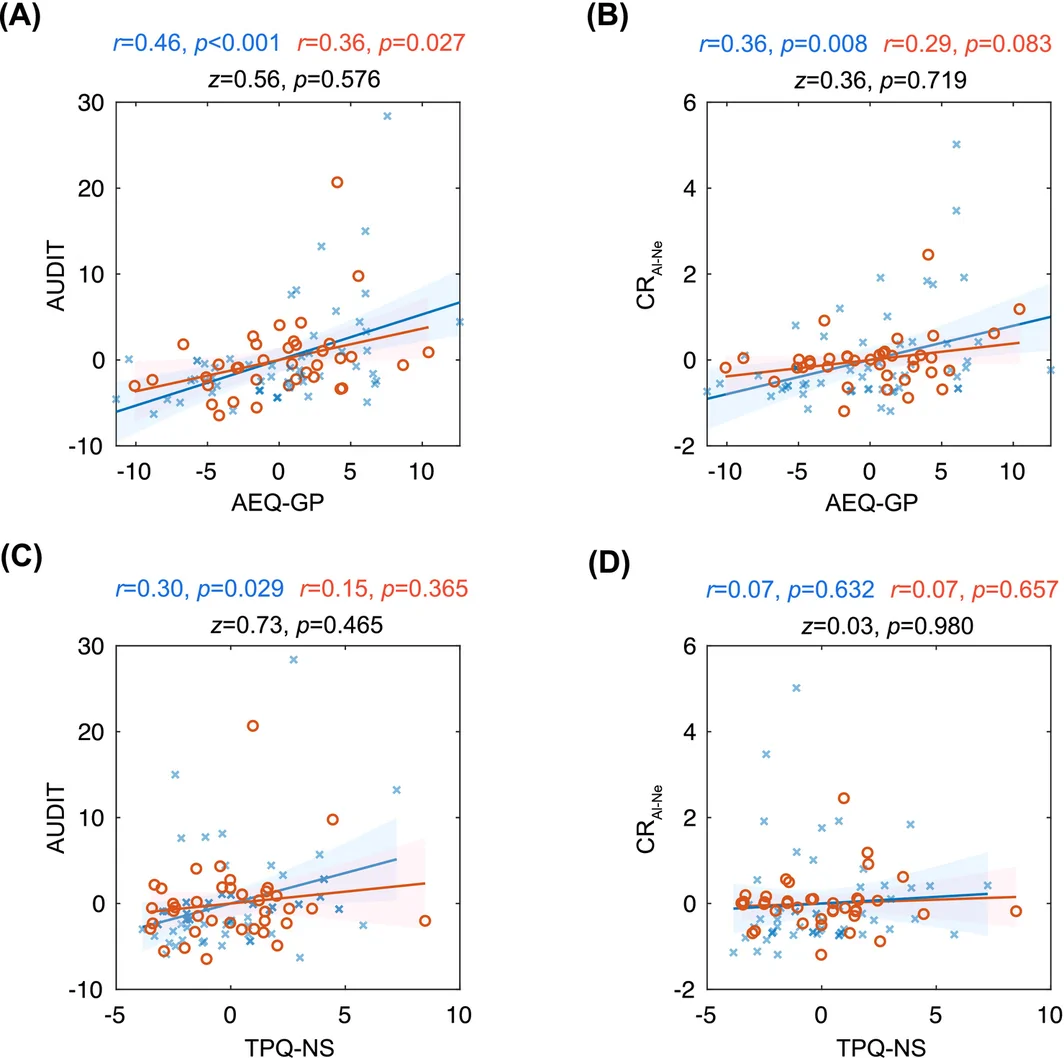
Scatter plots and linear regression of (A) AUDIT scores and (B) CR_Al‐Ne_ versus AEQ‐GP scores; and (C) AUDIT scores and (D) CR_Al‐Ne_ versus TPQ‐NS scores for males (blue cross) and females (red circle) separately. The *r* and *p* values of the linear regressions (males/females in blue/red) as well as the *z* and *p* values of slope test of males versus females (black) are shown on the top of each panel. None of the slope tests were significant. AEQ‐GP, Alcohol Expectancy Questionnaire–Global Positive; CR_Al‐Ne_, craving ratings during ‘alcohol>neutral’ blocks; TPQ‐NS, Tridimensional Personality Questionnaire–Novelty Seeking. Residuals are plotted here from the regression with age as a covariate. Solid lines and shades represent the regressions and 95% confidence intervals, respectively.

### 3.2 Skin Conductance Response to Alcohol Cue Exposure

Males showed an SCR (mean ± SD) of 0.23 ± 0.31 and 0.27 ± 0.34 μS in alcohol and neural blocks, respectively. Females showed an SCR of 0.37 ± 0.56 and 0.28 ± 0.37 μS in alcohol and neural blocks, respectively. A two‐by‐two repeated‐measures ANOVA with age as a covariate showed no significant main effect of sex (*F* = 0.42, *p* = 0.520) or of cue block (*F* = 0.41, *p* = 0.524), but a significant interaction effect (*F* = 4.00, *p* = 0.049).

In a within‐subject contrast of SCR during ‘alcohol>neutral’ blocks, males showed an SCR_Al‐Ne_ (mean ± SD) of −0.04 ± 0.28 μS and females showed an SCR_Al‐Ne_ of 0.09 ± 0.34 μS. Females relative to males showed a significantly higher SCR_Al‐Ne_, with age as a covariate (*F* = 3.99, *p* = 0.049). The SCRs are plotted for all subjects and for males and females separately in Figure 2.

**FIGURE 2:**
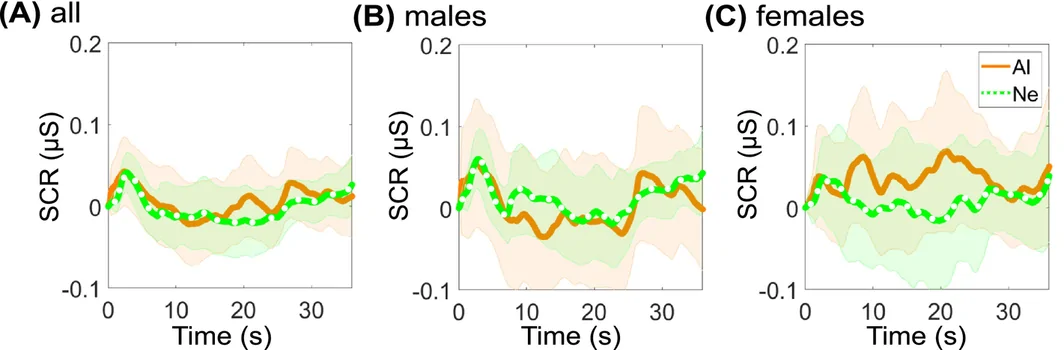
Skin conductance response (SCR; in μS) of alcohol (Al; orange) and neutral (Ne; green) blocks for (a) all, (b) males and (c) females. Lines and shades show the values of mean and SD, respectively.

In a linear regression, males showed a significantly positive correlation of SCR_Al‐Ne_ with AUDIT scores (*r* = 0.27, *p* = 0.048) whereas females showed a negative, though not significant, correlation (*r* = −0.18, *p* = 0.267), and a slope test confirmed the sex differences (*z* = 2.12, *p* = 0.034), all with age as a covariate (Figure 3A). Similarly, slope tests confirmed sex differences in the correlations of SCR_Al‐Ne_ with AEQ‐GP (males: *r* = 0.30, *p* = 0.030; females: *r* = −0.13, *p* = 0.432; slope test *z* = 2.03, *p* = 0.042; Figure 3B) and with TPQ‐NS (males: *r* = 0.28, *p* = 0.038; females: *r* = −0.17, *p* = 0.296; slope test *z* = 2.12, *p* = 0.034; Figure 3C) scores. However, SCR_Al‐Ne_ was not significantly correlated with CR_Al‐Ne_ in males (*r* = −0.02, *p* = 0.891) or in females (*r* = 0.07, *p* = 0.693). It should be noted that, although the tests for these associations were specified a priori, these slope differences were no longer significant after correcting for multiple comparisons (*p*'s > 0.05/4 = 0.0125).

**FIGURE 3:**
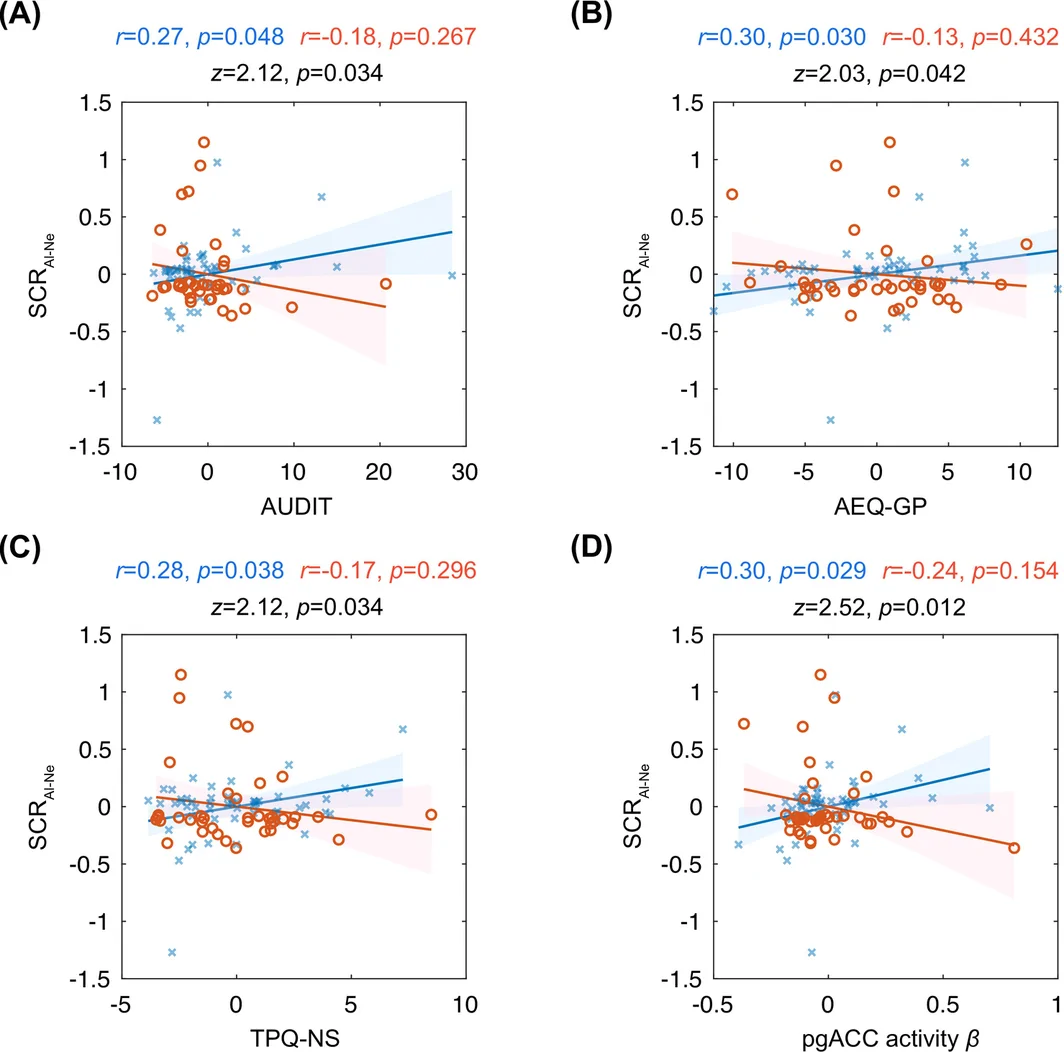
Scatter plots and linear regression of the differences in skin conductance response in ‘alcohol>neutral’ blocks (SCR_Al‐Ne_, μS) versus (A) AUDIT scores, (B) Alcohol Expectancy Questionnaire–Global Positive (AEQ‐GP) scores, (C) Tridimensional Personality Questionnaire–Novelty Seeking (TPQ‐NS) scores, and (D) activity *β* of the pregenual anterior cingulate cortex (pgACC) for males (blue cross) and females (red circle) separately. Residuals are plotted here from the regression with age as a covariate. Solid lines and shades represent the regressions and 95% confidence intervals, respectively. Correlation coefficients *r*, slope tests *z*‐scores, and corresponding *p* values are presented on the top of each subplot.

### 3.3 Brain Imaging

#### 3.3.1 Regional Responses to Alcohol>Neutral Cues

Regional brain responses to ‘alcohol>neutral’ cue exposure of all subjects and of males and females alone are shown in Figure S1. At voxel *p* < 0.001, uncorrected, the clusters included bilateral mPFC, occipital cortex, cerebellum, hippocampus, caudate, superior frontal gyri (SFG), predominantly in the left hemisphere and supplementary motor area (SMA) in all subjects; bilateral mPFC (predominantly left‐hemispheric), occipital cortex, cerebellum, amygdala, parahippocampal gyri, hippocampus, insula, thalamus, mid‐cingulate cortex, SFG (predominantly left‐hemispheric) and SMA in males alone; and a smaller cluster of bilateral mPFC and right occipital regions in females alone. In a two‐sample *t*‐test, males and females did not show any voxels with significant sex differences at the same threshold.

#### 3.3.2 Regional Responses to Alcohol>Neutral Cues in Correlation With AUDIT Scores

Whole‐brain regression of the contrast of ‘alcohol>neutral’ showed that, with age as a covariate, greater activity of bilateral mPFC, in the area of pgACC, was significantly correlated with higher AUDIT scores in all subjects (Figure 4A; 1 cluster: size *k* = 972, MNI coordinates [−8, 34, 11], peak voxel *Z* = 4.59) and, at the same threshold, in males (Figure 4B; 1 cluster: size *k* = 1456, MNI coordinates [3, 22, −2], peak voxel *Z* = 5.24) but not in females (Figure 4C) alone.

**FIGURE 4:**
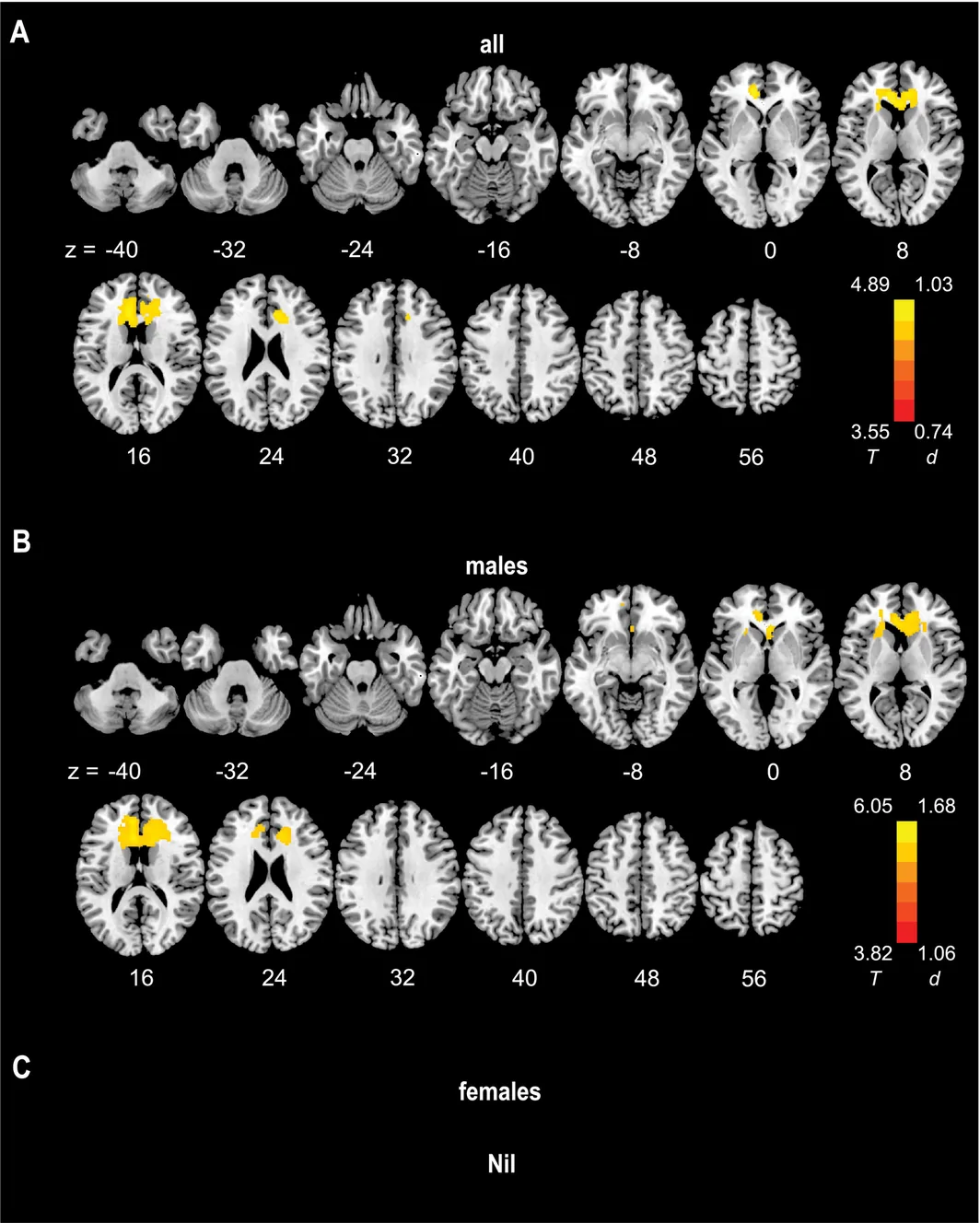
A voxelwise whole brain regression of ‘alcohol>neutral’ contrast on AUDIT scores, with age as a covariate, in (A) all, (B) males and (C) females. The results were evaluated at voxel *p* < 0.001 uncorrected, in combination with cluster *p* < 0.05 family‐wise error rate (FWE) corrected. Warm represents positive correlation. No negative correlation was identified. Colour bars show voxel *T* and Cohen's *d* values. Nil, no significant findings.

We also performed supplementary whole‐brain regression analyses examining alcohol and neutral cue responses separately. Interestingly, no significant associations between alcohol cue responses and AUDIT scores were observed, whereas responses during neutral cue blocks showed significant negative associations with AUDIT scores in all subjects and males only (Figure S2).

As described in the Methods, there were fewer females in the sample and the pgACC cluster could have just missed the threshold; thus, we conducted a slope test to examine sex difference. The slope test confirmed significant sex differences in the correlations between pgACC activity *β* and AUDIT scores (males: *r* = 0.62, *p* < 0.001; females: *r* = 0.17, *p* = 0.307; *z* = 2.54, *p* = 0.011). Moreover, as shown in Figure 3D, the SCR_Al‐Ne_ was also significantly correlated with pgACC activity *β* in males (*r* = 0.30, *p* = 0.029), but not in females (*r* = −0.24, *p* = 0.154), with the slope test confirming sex difference (*z* = 2.52, *p* = 0.012). Note that the slope difference remained significant after correcting for multiple comparisons (*p <* 0.05/4 = 0.0125). Furthermore, in a block‐by‐sex repeated‐measures ANOVA of pgACC activity *β* with age as a covariate, we did not observe any significant main effect of block (*F* = 0.45, *p* = 0.503) or sex (*F* = 1.08, *p* = 0.301) or block‐by‐sex interaction effect (*F* = 0.87, *p* = 0.353). We did not identify cue reactivity of any clusters in significant correlation with AEQ‐GP, TPQ‐NS scores or SCR_Al‐Ne_ in whole brain regressions evaluated at the same threshold.

### 3.4 Chain Mediation Model

To explore potential pathways linking physiological arousal, pgACC activity, alcohol expectancy and problem drinking severity, we evaluated a series of theoretically motivated chain mediation models. The only significant model showed SCR_Al‐Ne_➔pgACC activity *β*➔AEQ‐GP scores ➔AUDIT scores (Figure 5), where the indirect effect of SCR_Al‐Ne_ on AUDIT through AEQ‐GP scores alone was not significant. However, a significant serial indirect effect through pgACC activity *β* and AEQ‐GP scores was observed, suggesting that SCR_Al‐Ne_ influenced AUDIT scores via a sequential process in which pgACC activity *β* modulated AEQ‐GP scores.

**FIGURE 5:**
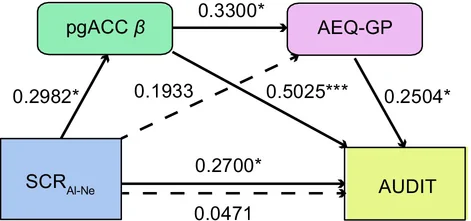
Chain mediation model. SCR_Al‐Ne_ influenced AUDIT scores via a sequential process in which pgACC activity *β* modulated AEQ‐GP scores, with age included as a covariate. Standardized coefficients are shown, with *p < 0.05 and ***p < 0.001. AEQ‐GP, Alcohol Expectancy Questionnaire–Global Positive; pgACC, perigenual anterior cingulate cortex; SCR_Al‐Ne_, skin conductance response in ‘alcohol>neutral’ blocks.

## 4 Discussion

We found that, although females as compared to males showed greater cue‐elicited physiological arousal, only males exhibited significant interindividual associations between cue‐evoked SCR and alcohol use severity, alcohol expectancy and novelty seeking trait. Alcohol cue exposure engaged the pgACC in association with problem drinking severity and alcohol expectancy in males but not in females. Furthermore, mediation modelling showed that pgACC response and alcohol expectancy interlink cue‐evoked SCR and problem drinking severity. Together, these findings suggest differences in physiological and neural responses to alcohol cues and how these responses relate to individual behavioural traits differently between males and females. We discussed the main findings below.

### 4.1 Physiological Arousal and Drinking Severity, Alcohol Expectancy and Novelty Seeking: Sex‐Specific Associations

SCR provides an objective index of sympathetic arousal and has been widely used to capture automatic, cue‐elicited motivational processes [43, 44]. Although both males and females showed comparable SCRs during alcohol and neutral blocks, females exhibited a significantly greater SCR contrast between blocks than males. This finding is broadly consistent with prior evidence suggesting heightened autonomic or emotional reactivity to affective cues in females [45]. However, this greater physiological reactivity did not appear to associate significantly with problem drinking severity or related behavioural traits across females in this sample.

In males, in contrast, higher cue‐elicited physiological arousal was associated with more severe problem drinking, and greater positive alcohol expectancies and novelty seeking. These convergent associations suggest that, in males, autonomic arousal during alcohol cue exposure indexes motivational salience in link with drinking‐related beliefs and approach behavioural traits. This pattern aligns with models of incentive sensitization, in which repeated alcohol use enhances the motivational value of alcohol‐related cues, leading to heightened physiological and behavioural responses that promote drinking [46].

Neuroimaging studies have demonstrated that alcohol cue reactivity in males is more strongly coupled with mesocorticolimbic, including ventral striatal and medial prefrontal cortical, circuit activities [47], which are implicated in reward valuation and reinforcement learning. In parallel, psychophysiological studies showed that sympathetic arousal, as reflected in SCR, is closely related to approach motivational states and dopaminergic signalling within reward circuits [24]. Together, these findings suggest that, in males, heightened SCR during alcohol cue exposures reflects a reward‐driven motivational process. The sex‐different coupling between autonomic arousal and reward‐seeking traits is consistent with a literature of predominantly reward‐driven drinking behaviour in males [48]. Although females showed higher SCR_Al‐Ne_ on average, significant associations with drinking severity or related behavioural traits were not observed. This pattern may indicate differences in the mechanisms linking physiological arousal to drinking behaviour between the sexes; however, it may also reflect the smaller sample size and reduced statistical power in females. Larger studies are needed to distinguish between these possibilities.

Notably, our findings appear to be different from an earlier report where AUD participants with lower drinking severity, but not those with higher severity, had elevated anticipatory SCRs during the Iowa gambling task [24]. The absence of elevated SCR in individuals with more severe AUD was interpreted as reflecting deficits in interoceptive processing. One possible explanation is that our sample comprised predominantly nondependent drinkers with relatively low to moderate AUDIT scores, in whom interoceptive signalling and its coupling with reward valuation may remain intact or even sensitized. In contrast, in people with more severe AUD, chronic alcohol use may have blunted interoceptive signalling or disrupted its integration with cortical valuation systems. Taken together, these findings may suggest a stage‐dependent relationship between physiological arousal and drinking severity, in which heightened interoceptive signalling characterizes earlier stages of drinking, whereas diminished or dysregulated interoception emerges with greater severity and chronicity of alcohol consumption.

### 4.2 pgACC Reactivity Links Cue‐Elicited Arousal and Problem Drinking Severity in Males

Alcohol cues elicited robust activation in a wide swath of mPFC regions across all subjects, consistent with prior work implicating the mPFC in cue valuation, salience attribution and decision‐making [49, 50]. Whole‐brain regression analyses revealed that, of the mPFC, a smaller cluster in the pgACC showed activity in positive correlation with AUDIT scores in males but not in females, and slope tests confirmed the sex difference. The pgACC occupies a central position in neural networks supporting valuation, affective regulation and interoceptive integration [51]. It receives viscerosensory inputs and is well positioned to translate bodily arousal into motivational states to guide behaviours [52]. The positive association between pgACC activation and problem drinking severity may reflect engagement of neural processes linking interoceptive signals with reward‐related valuation during alcohol cue exposure [53].

Notably, we did not observe sex differences in alcohol cue‐evoked activation in independent sample *t*‐test of males versus females, underscoring the importance of addressing individual differences in behaviour and biology. The current findings suggest that sex differences in alcohol cue reactivity may manifest more clearly at the level of brain‐behaviour and brain–body relationships rather than group mean neural responses. Despite exhibiting greater physiological arousal to alcohol cues, females did not show significant associations between SCR_Al‐Ne_ and drinking severity, alcohol expectancy, novelty seeking or pgACC activation. Several interpretations are possible. First, physiological arousal in females may reflect a more generalized affective or contextual response to cue stimuli, rather than alcohol‐specific incentive salience. Second, females may draw more heavily on other psychological processes, such as stress reactivity, that were not captured by the measures included in the present study. It is also possible that hormonal fluctuations, unmeasured psychosocial factors or nonlinear relationships between arousal and behaviour contribute to greater heterogeneity in females [54]. Thus, the absence of significant associations should not be interpreted as evidence that comparable physiological or neural mechanisms do not exist in females. Rather, these findings indicate that such relationships were not detected in the current sample. The relatively smaller female sample, lower alcohol use severity, and greater heterogeneity among females may have reduced the statistical power needed to detect comparable associations. In addition, the follow‐up analyses focused on the pgACC identified from males, which may have further limited sensitivity to identify neural correlates in females. Future studies with larger, more balanced samples are needed to determine whether similar or distinct relationships among physiological arousal, neural cue reactivity and alcohol‐related behaviours manifest in females.

Exploratory chain mediation analyses identified a pathway in which pgACC reactivity and alcohol expectancy interlinked SCR_Al‐Ne_ and AUDIT scores. This finding suggests a pathway in which heightened sympathetic arousal to ‘alcohol>neutral’ cues (i.e., SCR_Al‐Ne_) is associated with greater problem drinking severity through pgACC cue response and a behavioural trait motivating alcohol use. As described earlier, the pgACC has been implicated in valuation, self‐referential processing and integration of interoceptive signals with motivational salience [15]. Thus, these findings align with models of addiction that emphasize the role of prefrontal‐interoceptive integration in sustaining substance misuse [55]. In males, heightened physiological reactivity may translate into more severe problem alcohol use through pgACC cue activity directly and/or indirectly through its impact on alcohol expectancy. This pathway may explain interindividual variability in the extent to which elevated autonomic reactivity translates into problem drinking. Because multiple candidate mediation models were evaluated using cross‐sectional data, these findings should be considered hypothesis‐generating and require replication in independent longitudinal or experimental studies.

### 4.3 Supplementary Findings and Methodological Considerations

To address the potential issue regarding subtraction‐based contrasts [56], we performed supplementary analyses examining alcohol and neutral cue responses separately. Interestingly, across all subjects and in males only, no significant associations between alcohol cue responses and AUDIT scores were observed. In contrast, neural cue responses showed negative associations with AUDIT scores. Together, these findings suggest that the observed relationship for the alcohol>neutral contrast is driven primarily by reduced neural responses to neutral cues rather than enhanced responses to alcohol cues. This finding seems consistent with models of addiction where increasing severity of alcohol misuse is accompanied not only by altered processing of alcohol‐related stimuli but also by diminished responsiveness to nondrug stimuli, thereby increasing the relative salience of alcohol cues.

An additional methodological consideration concerns the ROI analyses. The pgACC ROI was identified from the whole‐brain regression analysis and subsequently used to characterize relationships with physiological and behavioural measures within the same dataset. Consequently, these ROI analyses were not statistically independent and may have yielded inflated effect size estimates. Furthermore, because the ROI was derived from the male whole‐brain findings, the follow‐up analyses may have been more sensitive to detecting associations in males than in females. Therefore, the present findings are best interpreted as identifying pgACC‐behaviour relationships in males within this sample rather than providing definitive evidence for sex differences in these neural mechanisms. Independent replication using larger samples and independently defined ROIs will be important to establish the robustness and generalizability of these findings.

Notably, the sample spanned a broad age range (19–85 years), encompassing individuals at markedly different stages of neurobiological, autonomic and behavioural development. Aging is associated with changes in sympathetic nervous system function, interoceptive processing and neural responses to motivationally salient stimuli, all of which may influence SCR and alcohol cue reactivity [57, 58, 59]. In addition, patterns of alcohol use, alcohol expectancies and motivations for drinking can differ substantially across the lifespan [60, 61, 62]. Although age was included as a covariate in all analyses, statistical adjustment may not fully account for potentially nonlinear or interactive effects of aging on physiological and neural cue responses. Therefore, the current findings should be interpreted as reflecting associations that generalized across a heterogeneous adult sample. Future studies with larger samples should examine age‐specific effects and potential interactions of age and sex in alcohol cue reactivity.

### 4.4 Limitations and Conclusions

Several limitations should be considered when interpreting these findings. First, the sample comprised predominantly community‐recruited adults with relatively low to moderate AUDIT scores, and only a small proportion met criteria for AUD, particularly among females. Consequently, the observed associations amongst physiological arousal, pgACC activity and alcohol‐related behavioural measures may primarily reflect lower‐severity drinkers and may not generalize to individuals with more severe or treatment‐seeking AUD. Because cue reactivity is known to vary with the severity and chronicity of alcohol use, future studies should examine these relationships across a broader spectrum of alcohol use severity. Second, the sample size for females was smaller than for males, potentially reducing statistical power to detect brain‐behaviour or brain‐physiological associations in females. Because multiple behavioural correlation and slope analyses were conducted, some findings, particularly those with marginal *p* values, should be interpreted cautiously until replicated in larger independent samples. Third, these findings were observed across a broad adult age range, suggesting that the identified relationships may be robust to age‐related variability. However, future work is needed to determine whether the observed sex‐specific associations differ across age groups. Fourth, despite the mediation findings, the cross‐sectional design precludes causal inference regarding “the chain of events” leading to problem alcohol use. Fifth, we did not assess menstrual cycle phase or circulating sex hormones, which may influence physiological and neural responses to alcohol cues in females. In addition, we did not formally examine habituation of SCR across repeated cue blocks. Future studies should evaluate temporal changes in physiological responses over the course of cue exposure. Finally, although only a small number of participants were smokers and none exhibited severe nicotine dependence, tobacco/nicotine use remains a potential confound given the well‐established comorbidity between alcohol and nicotine use [63, 64] and the known effects of nicotine on autonomic arousal, reward processing and cue reactivity. Future studies should more comprehensively assess and control for nicotine use when examining alcohol cue reactivity.

In sum, the present study provides evidence linking physiological arousal, neural responses during alcohol cue exposure and behavioural traits in a sex‐specific manner. Our findings indicate that, within the present sample, alcohol cues elicited coordinated autonomic and pgACC responses associated with motivational traits and problem drinking severity in males. Comparable relationships were not observed in females. These findings should not be interpreted as evidence that similar mechanisms are absent in females but rather that they were not detected in the current dataset. Future studies with larger and more representative samples are needed to determine whether the observed associations reflect true sex differences or differences in statistical power, drinking severity or other methodological factors. In addition, these findings should be interpreted cautiously given the nonindependent ROI analyses and require replication using independent samples and prospectively defined ROIs before stronger conclusions regarding sex differences can be drawn.

Together, these results highlight the importance of integrating physiological, neural and behavioural measures to understand individual and sex differences in alcohol cue reactivity. Elucidating distinct cue‐response pathways in males and females may inform more targeted prevention and intervention strategies to disrupt cue‐driven drinking behaviour.

## Author Contributions

**Yu Chen:** writing – review and editing, writing – original draft, visualization, methodology, formal analysis, data curation, conceptualization. **Hak Kei Wong:** writing – review and editing, writing – original draft, formal analysis. **Shefali Chaudhary:** writing – review and editing, writing – original draft, methodology, formal analysis. **Herta H. Chao:** writing – review and editing, writing – original draft, supervision, methodology, conceptualization. **Sheng Zhang:** writing – review and editing, writing – original draft, formal analysis, data curation. **Chiang‐Shan R. Li:** writing – review and editing, writing – original draft, supervision, methodology, funding acquisition, conceptualization.

## Funding

This work was supported by the National Institutes of Health (AG072893).

## Ethics Statement

The study was conducted according to a protocol approved by the Institutional Review Board of Yale University. Written informed consent was obtained from all subjects prior to their participation.

## Conflicts of Interest

The authors declare no conflicts of interest.

## Supporting information


**Figure S1:** Regional brain activations to ‘alcohol>neutral’ blocks for (A) all, (B) males and (C) females. The results were evaluated at voxel *p* < 0.001 uncorrected, in combination with cluster *p* < 0.05, family‐wise error rate (FWE) corrected. Warm colour represents the contrast of Alcohol>Neutral. No significant findings for the contrast of ‘neutral>alcohol.’ Colour bars show voxel *T* and Cohen's *d* values.
**Figure S2:** A voxelwise whole brain regression of ‘Neutral’ contrast on AUDIT scores, with age as a covariate, in (A) all, (B) males and (C) females. The results were evaluated at voxel p < 0.001 uncorrected, in combination with cluster p < 0.05 family‐wise error rate (FWE) corrected. Cool colour represents negative correlation. No positive correlation was identified. Color bars show voxel T and Cohen's d values. Nil, no significant findings.

## Data Availability

Data will be made available on request.

## References

[1] D. O'Tousa and N. Grahame , “Habit Formation: Implications for Alcoholism Research,” Alcohol 48, no. 4 (2014): 327–335. 10.1016/j.alcohol.2014.02.004 PMC4096986 24835007

[2] C. B. Kohen , R. U. Cofresí , B. D. Bartholow , and T. M. Piasecki , “Alcohol Craving in the Natural Environment: Moderating Roles of Cue Exposure, Drinking, and Alcohol Sensitivity,” Experimental and Clinical Psychopharmacology 31, no. 1 (2023): 57–71. 10.1037/pha0000540 PMC9276840 35025586

[3] R. Green , E. Grodin , A. C. Lim , et al., “The Interplay Between Subjective Response to Alcohol, Craving, and Alcohol Self‐Administration in the Human Laboratory,” Alcoholism, Clinical and Experimental Research 43, no. 5 (2019): 907–915. 10.1111/acer.14001 PMC6519957 30860603

[4] C. A. Motschman , M. Amlung , and D. M. McCarthy , “Alcohol Demand as a Predictor of Drinking Behavior in the Natural Environment,” Addiction 117, no. 7 (2022): 1887–1896. 10.1111/add.15822 PMC10061588 35112741

[5] J. P. Schacht , R. F. Anton , and H. Myrick , “Functional Neuroimaging Studies of Alcohol Cue Reactivity: A Quantitative Meta‐Analysis and Systematic Review,” Addiction Biology 18, no. 1 (2013): 121–133. 10.1111/j.1369-1600.2012.00464.x PMC3419322 22574861

[6] J. Zeng , S. Yu , H. Cao , Y. Su , Z. Dong , and X. Yang , “Neurobiological Correlates of Cue‐Reactivity in Alcohol‐Use Disorders: A Voxel‐Wise Meta‐Analysis of fMRI Studies,” Neuroscience and Biobehavioral Reviews 128 (2021): 294–310. 10.1016/j.neubiorev.2021.06.031 34171325

[7] C. Pulido , S. A. Brown , K. Cummins , M. P. Paulus , and S. F. Tapert , “Alcohol Cue Reactivity Task Development,” Addictive Behaviors 35, no. 2 (2010): 84–90. 10.1016/j.addbeh.2009.09.006 PMC2783802 19800172

[8] S. Zhornitsky , S. Zhang , J. S. Ide , et al., “Alcohol Expectancy and Cerebral Responses to Cue‐Elicited Craving in Adult Nondependent Drinkers,” Biological Psychiatry: Cognitive Neuroscience and Neuroimaging 4, no. 5 (2019): 493–504. 10.1016/j.bpsc.2018.11.012 PMC6500759 30711509

[9] S. Chaudhary , Y. Chen , S. Zhornitsky , et al., “The Effects of Age on the Severity of Problem Drinking: Mediating Effects of Positive Alcohol Expectancy and Neural Correlates,” Addiction Biology 28, no. 6 (2023): e13278. 10.1111/adb.13278 37252876

[10] N. D. Volkow and R. D. Baler , “Brain Imaging Biomarkers to Predict Relapse in Alcohol Addiction,” JAMA Psychiatry 70, no. 7 (2013): 661–663. 10.1001/jamapsychiatry.2013.1141 PMC4827769 23636789

[11] K. Starcke , S. Antons , P. Trotzke , and M. Brand , “Cue‐Reactivity in Behavioral Addictions: A Meta‐Analysis and Methodological Considerations,” Journal of Behavioral Addictions 7, no. 2 (2018): 227–238. 10.1556/2006.7.2018.39 PMC6174580 29788752

[12] M. J. Robinson , T. E. Robinson , and K. C. Berridge , “Incentive Salience and the Transition to Addiction,” Biological Research on Addiction 2 (2013): 391–399.

[13] C. F. Zink , G. Pagnoni , J. Chappelow , M. Martin‐Skurski , and G. S. Berns , “Human Striatal Activation Reflects Degree of Stimulus Saliency,” NeuroImage 29, no. 3 (2006): 977–983. 10.1016/j.neuroimage.2005.08.006 PMC1819473 16153860

[14] C. Xia , A. Touroutoglou , K. S. Quigley , L. Feldman Barrett , and B. C. Dickerson , “Salience Network Connectivity Modulates Skin Conductance Responses in Predicting Arousal Experience,” Journal of Cognitive Neuroscience 29, no. 5 (2017): 827–836. 10.1162/jocn_a_01087 PMC5690982 27991182

[15] A. Fujimoto , E. A. Murray , and P. H. Rudebeck , “Interaction Between Decision‐Making and Interoceptive Representations of Bodily Arousal in Frontal Cortex,” Proceedings of the National Academy of Sciences of the United States of America 118, no. 35 (2021): e2014781118. 10.1073/pnas.2014781118 PMC8536360 34452993

[16] A. Verdejo‐Garcia , L. Clark , and B. D. Dunn , “The Role of Interoception in Addiction: A Critical Review,” Neuroscience and Biobehavioral Reviews 36, no. 8 (2012): 1857–1869. 10.1016/j.neubiorev.2012.05.007 22659642

[17] G. G. Berntson and S. S. Khalsa , “Neural Circuits of Interoception,” Trends in Neurosciences 44, no. 1 (2021): 17–28. 10.1016/j.tins.2020.09.011 PMC8054704 33378653

[18] C. B. Padula , L.‐T. Tenekedjieva , D. M. McCalley , et al., “Targeting the Salience Network: A Mini‐Review on a Novel Neuromodulation Approach for Treating Alcohol Use Disorder,” Frontiers in Psychiatry 13 (2022): 893833. 10.3389/fpsyt.2022.893833 PMC9152026 35656355

[19] S. Zhang , S. Hu , H. H. Chao , X. Luo , O. M. Farr , and C. R. Li , “Cerebral Correlates of Skin Conductance Responses in a Cognitive Task,” NeuroImage 62, no. 3 (2012): 1489–1498. 10.1016/j.neuroimage.2012.05.036 PMC3408848 22634217

[20] W. Wang , S. Zhornitsky , T. M. Le , et al., “Cue‐Elicited Craving, Thalamic Activity, and Physiological Arousal in Adult Non‐Dependent Drinkers,” Journal of Psychiatric Research 116 (2019): 74–82. 10.1016/j.jpsychires.2019.06.005 PMC6606341 31202048

[21] F. Nees , C. Diener , M. N. Smolka , and H. Flor , “The Role of Context in the Processing of Alcohol‐Relevant Cues,” Addiction Biology 17, no. 2 (2012): 441–451. 10.1111/j.1369-1600.2011.00347.x 21790904

[22] J. E. Cassisi , M. Delehant , J. S. Tsoutsouris , and J. Levin , “Psychophysiological Reactivity to Alcohol Advertising in Light and Moderate Social Drinkers,” Addictive Behaviors 23, no. 2 (1998): 267–274. 10.1016/s0306-4603(97)00038-5 9573431

[23] M. S. Reid , F. Flammino , A. Starosta , J. Palamar , and J. Franck , “Physiological and Subjective Responding to Alcohol Cue Exposure in Alcoholics and Control Subjects: Evidence for Appetitive Responding,” Journal of Neural Transmission 113, no. 10 (2006): 1519–1535. 10.1007/s00702-005-0439-5 16604310

[24] W. B. Logge , K. C. Morley , P. S. Haber , and A. J. Baillie , “Impaired Decision‐Making and Skin Conductance Responses Are Associated With Reward and Punishment Sensitivity in Individuals With Severe Alcohol Use Disorder,” Neuropsychobiology 82, no. 2 (2023): 117–129. 10.1159/000529156 PMC10233679 36812895

[25] S. Zhang , S. Hu , J. Hu , P.‐L. Wu , H. H. Chao , and C.‐s R. Li , “Barratt Impulsivity and Neural Regulation of Physiological Arousal,” PLoS ONE 10, no. 6 (2015): e0129139. 10.1371/journal.pone.0129139 PMC4469608 26079873

[26] T. L. Verplaetse , K. P. Cosgrove , J. Tanabe , and S. A. McKee , “Sex/Gender Differences in Brain Function and Structure in Alcohol Use: A Narrative Review of Neuroimaging Findings Over the Last 10 Years,” Journal of Neuroscience Research 99, no. 1 (2021): 309–323. 10.1002/jnr.24625 PMC7584757 32333417

[27] T. M. Chaplin , K. Hong , K. Bergquist , and R. Sinha , “Gender Differences in Response to Emotional Stress: An Assessment Across Subjective, Behavioral, and Physiological Domains and Relations to Alcohol Craving,” Alcoholism, Clinical and Experimental Research 32, no. 7 (2008): 1242–1250. 10.1111/j.1530-0277.2008.00679.x PMC2575018 18482163

[28] A. M. Kaag , R. W. Wiers , T. J. de Vries , T. Pattij , and A. E. Goudriaan , “Striatal Alcohol Cue‐Reactivity Is Stronger in Male Than Female Problem Drinkers,” European Journal of Neuroscience 50, no. 3 (2019): 2264–2273. 10.1111/ejn.13991 PMC6767856 29888821

[29] M. Radoman , N. Fogelman , C. Lacadie , D. Seo , and R. Sinha , “Neural Correlates of Stress and Alcohol Cue‐Induced Alcohol Craving and of Future Heavy Drinking: Evidence of Sex Differences,” American Journal of Psychiatry 181, no. 5 (2024): 412–422. 10.1176/appi.ajp.20230849 PMC11117176 38706332

[30] M. R. Peltier , T. L. Verplaetse , Y. S. Mineur , et al., “Sex Differences in Stress‐Related Alcohol Use,” Neurobiology of Stress 10 (2019): 100149. 10.1016/j.ynstr.2019.100149 PMC6430711 30949562

[31] H. K. Wong , S. Chaudhary , Y. Chen , J. S. Ide , S. Zhang , and C.‐S. R. Li , “Cingulate and Frontopolar Cortical Projections to the Cerebellar Vermis Support Prolonged Reaction Time in Identifying Negative Emotional Scenes in Women,” NeuroImage 319 (2025): 121435. 10.1016/j.neuroimage.2025.121435 PMC12462808 40882825

[32] M. B. First , “Structured Clinical Interview for DSM‐IV Axis I Disorders Biometrics Research Department,” (1995).

[33] M. J. Bohn , T. F. Babor , and H. R. Kranzler , “The Alcohol Use Disorders Identification Test (AUDIT): Validation of a Screening Instrument for Use in Medical Settings,” Journal of Studies on Alcohol 56, no. 4 (1995): 423–432. 10.15288/jsa.1995.56.423 7674678

[34] J. B. Saunders , O. G. Aasland , T. F. Babor , J. R. De la Fuente , and M. Grant , “Development of the Alcohol Use Disorders Identification Test (AUDIT): WHO Collaborative Project on Early Detection of Persons With Harmful Alcohol Consumption‐II,” Addiction 88, no. 6 (1993): 791–804. 10.1111/j.1360-0443.1993.tb02093.x 8329970

[35] W. H. George , M. R. Frone , M. L. Cooper , M. Russell , J. B. Skinner , and M. Windle , “A Revised Alcohol Expectancy Questionnaire: Factor Structure Confirmation, and Invariance in a General Population Sample,” Journal of Studies on Alcohol 56, no. 2 (1995): 177–185. 10.15288/jsa.1995.56.177 7760564

[36] C. R. Cloninger , T. R. Przybeck , and D. M. Svrakic , “The Tridimensional Personality Questionnaire: US Normative Data,” Psychological Reports 69, no. 3 (1991): 1047–1057. 10.2466/pr0.1991.69.3.1047 1784653

[37] A. Kampov‐Polevoy , G. Bobashev , and J. C. Garbutt , “Exploration of the Impact of Combining Risk Phenotypes on the Likelihood of Alcohol Problems in Young Adults,” Alcohol and Alcoholism 57, no. 3 (2022): 357–363. 10.1093/alcalc/agab049 PMC9086793 34272558

[38] T. M. Le , Y. Chen , S. Chaudhary , and C. R. Li , “Problem Drinking and the Interaction of Reward, Negative Emotion, and Cognitive Control Circuits During Cue‐Elicited Craving,” Addiction Neuroscience 1 (2022): 100004.

[39] B. Figner , R. O. Murphy , M. Schulte‐Mecklenbeck , A. Kuehberger , and R. Ranyard , “Using Skin Conductance in Judgment and Decision Making Research,” in A Handbook of Process Tracing Methods for Decision Research (Psychology Press, 2011).

[40] B. Figner and R. O. Murphy , “Using Skin Conductance in Judgment and Decision Making Research,” in A Handbook of Process Tracing Methods for Decision Research: A Critical Review and User’s Guide (Psychology Press, 2011), 163–184.

[41] J. H. Zar , Biostatistical Analysis (Pearson Education India, 1999).

[42] Y. Chen , X. Luo , H.‐T. Li , G. Li , J. Ide , and C. R. Li , “The Effects of Polygenic Risks for Alcohol Misuse on Negative Emotion Processing in Young Adult Binge Drinkers,” Translational Psychiatry 15 (2025): 495. 10.1038/s41398-025-03719-3 PMC12638819 41271663

[43] C. Tong , D. H. Bovbjerg , and J. Erblich , “Smoking‐Related Videos for Use in Cue‐Induced Craving Paradigms,” Addictive Behaviors 32, no. 12 (2007): 3034–3044. 10.1016/j.addbeh.2007.07.010 PMC2078201 17725931

[44] S. D. LaRowe , M. E. Saladin , M. J. Carpenter , and H. P. Upadhyaya , “Reactivity to Nicotine Cues Over Repeated Cue Reactivity Sessions,” Addictive Behaviors 32, no. 12 (2007): 2888–2899. 10.1016/j.addbeh.2007.04.025 PMC4737433 17537583

[45] H.‐F. Hsieh , H.‐T. Hsu , P.‐C. Lin , et al., “The Effect of Age, Gender, and Job on Skin Conductance Response Among Smartphone Users Who Are Prohibited From Using Their Smartphone,” International Journal of Environmental Research and Public Health 17, no. 7 (2020): 2313. 10.3390/ijerph17072313 PMC7177482 32235441

[46] T. E. Robinson and K. C. Berridge , “The Incentive‐Sensitization Theory of Addiction 30 Years on,” Annual Review of Psychology 76 (2025): 29–58. 10.1146/annurev-psych-011624-024031 PMC11773642 39094061

[47] J. M. Barker and J. R. Taylor , “Sex Differences in Incentive Motivation and the Relationship to the Development and Maintenance of Alcohol Use Disorders,” Physiology and Behavior 203 (2019): 91–99. 10.1016/j.physbeh.2017.09.027 PMC5878117 28974459

[48] C. E. Ardinger , C. C. Lapish , C. L. Czachowski , and N. J. Grahame , “A Critical Review of Front‐Loading: A Maladaptive Drinking Pattern Driven by Alcohol's Rewarding Effects,” Alcoholism, Clinical and Experimental Research 46, no. 10 (2022): 1772–1782. 10.1111/acer.14924 PMC9588658 36239713

[49] C. A. Hanlon , L. T. Dowdle , N. B. Gibson , et al., “Cortical Substrates of Cue‐Reactivity in Multiple Substance Dependent Populations: Transdiagnostic Relevance of the Medial Prefrontal Cortex,” Translational Psychiatry 8, no. 1 (2018): 186. 10.1038/s41398-018-0220-9 PMC6128822 30194288

[50] J. M. Engelmann , F. Versace , J. D. Robinson , et al., “Neural Substrates of Smoking Cue Reactivity: A Meta‐Analysis of fMRI Studies,” NeuroImage 60, no. 1 (2012): 252–262. 10.1016/j.neuroimage.2011.12.024 PMC3288122 22206965

[51] N. Palomero‐Gallagher , F. Hoffstaedter , H. Mohlberg , S. B. Eickhoff , K. Amunts , and K. Zilles , “Human Pregenual Anterior Cingulate Cortex: Structural, Functional, and Connectional Heterogeneity,” Cerebral Cortex 29, no. 6 (2019): 2552–2574. 10.1093/cercor/bhy124 PMC6519696 29850806

[52] K. S. Taylor , D. A. Seminowicz , and K. D. Davis , “Two Systems of Resting State Connectivity Between the Insula and Cingulate Cortex,” Human Brain Mapping 30, no. 9 (2009): 2731–2745. 10.1002/hbm.20705 PMC6871122 19072897

[53] H. D. Critchley , “Neural Mechanisms of Autonomic, Affective, and Cognitive Integration,” Journal of Comparative Neurology 493, no. 1 (2005): 154–166. 10.1002/cne.20749 16254997

[54] A. Erol , A. M. C. Ho , S. J. Winham , and V. M. Karpyak , “Sex Hormones in Alcohol Consumption: A Systematic Review of Evidence,” Addiction Biology 24, no. 2 (2019): 157–169. 10.1111/adb.12589 PMC6585852 29280252

[55] M. P. Paulus , “Interoceptive Processing in Substance Use Disorders (SUDs): From the Neuroanatomy to Insights From Computational Models and Predictive Coding Frameworks,” in The Bodily Self, Emotion, and Subjective Time: Exploring Interoception Through the Contributions of AD (Bud) Craig (Springer Nature Switzerland, 2024), 159–178. 10.1007/7854_2024_519 39587011

[56] P. Bach , I. Reinhard , A. Koopmann , et al., “Test–Retest Reliability of Neural Alcohol Cue‐Reactivity: Is There Light at the End of the Magnetic Resonance Imaging Tube?,” Addiction Biology 27, no. 1 (2022): e13069. 10.1111/adb.13069 34132011

[57] M. Qi , X. Shen , Y. Zeng , X. Lin , M. Suliman , and P. Li , “Interoception and Mental Health in Middle‐Aged and Elderly Adults: A Systematic Review and Meta‐Analysis,” Neuroscience and Biobehavioral Reviews 172 (2025): 106104. 10.1016/j.neubiorev.2025.106104 40081440

[58] J. Murphy , H. Geary , E. Millgate , C. Catmur , and G. Bird , “Direct and Indirect Effects of Age on Interoceptive Accuracy and Awareness Across the Adult Lifespan,” Psychonomic Bulletin and Review 25, no. 3 (2018): 1193–1202. 10.3758/s13423-017-1339-z PMC5990557 28685271

[59] M. Mather , “The Affective Neuroscience of Aging,” Annual Review of Psychology 67, no. 1 (2016): 213–238. 10.1146/annurev-psych-122414-033540 PMC5780182 26436717

[60] E. V. Sullivan and A. Pfefferbaum , “Brain‐Behavior Relations and Effects of Aging and Common Comorbidities in Alcohol Use Disorder: A Review,” Neuropsychology 33, no. 6 (2019): 760–780. 10.1037/neu0000557 PMC7461729 31448945

[61] D. Satre and B. Knight , “Alcohol Expectancies and Their Relationship to Alcohol Use: Age and Sex Differences,” Aging & Mental Health 5, no. 1 (2001): 73–83. 10.1080/13607860020020672 11513017

[62] D. Heim , R. L. Monk , and A. W. Qureshi , “An Examination of the Extent to Which Drinking Motives and Problem Alcohol Consumption Vary as a Function of Deprivation, Gender and Age,” Drug and Alcohol Review 40, no. 5 (2021): 817–825. 10.1111/dar.13221 33244870

[63] L. L. Hurley , R. E. Taylor , and Y. Tizabi , “Positive and Negative Effects of Alcohol and Nicotine and Their Interactions: A Mechanistic Review,” Neurotoxicity Research 21, no. 1 (2012): 57–69. 10.1007/s12640-011-9275-6 PMC3377362 21932109

[64] D. E. Falk , H.‐y. Yi , and S. Hiller‐Sturmhöfel , “An Epidemiologic Analysis of Co‐Occurring Alcohol and Tobacco Use and Disorders: Findings From the National Epidemiologic Survey on Alcohol and Related Conditions,” Alcohol Research and Health 29, no. 3 (2006): 162. PMC6527037 17373404

