# Supplementary material for: Physiological Arousal During Alcohol Cue Exposure in Adult Drinkers: Sex Differences in Neural and Clinical Correlates: Alcohol Cue Exposure and Arousal
Source: Addict Biol. 2026 Nov 3;31(11):e70197. doi: 10.1111/adb.70197 (PMC13730815; doi:10.1111/adb.70197)
Supplement: Supplementary file 1 [file ADB-31-e70197-s001.docx]

**SUPPLEMENT:** Chen et al., Physiological arousal during alcohol cue exposure in adult drinkers: Sex differences in neural and clinical correlates

**Supplementary Figure S1.** Regional brain activations to ‘Alcohol>Neutral’ blocks for **(A)** all, **(B)** males, and **(C)** females. The results were evaluated at voxel *p*<0.001 uncorrected, in combination with cluster *p*<0.05, family-wise error rate (FWE) corrected. Warm color represents the contrast of Alcohol>Neutral. No significant findings for the contrast of ‘Neutral>Alcohol’. Color bars show voxel *T* and Cohen’s *d* values.

**Supplementary Figure S2.** A voxelwise whole brain regression of ‘Neutral’ contrast on AUDIT scores, with age as a covariate, in **(A)** all, **(B)** males, and **(C)** females. The results were evaluated at voxel *p*<0.001 uncorrected, in combination with cluster *p*<0.05 family-wise error rate (FWE) corrected. Cool color represents negative correlation. No positive correlation was identified. Color bars show voxel *T* and Cohen’s *d* values. Nil: no significant findings.
